# A Vision-Assisted Acoustic Channel Modeling Framework for Smartphone Indoor Localization

**DOI:** 10.3390/s26020717

**Published:** 2026-01-21

**Authors:** Can Xue, Huixin Zhuge, Zhi Wang

**Affiliations:** 1 State Key Laboratory of Industrial Control Technology, Zhejiang University, Hangzhou 310027, China; 2 Huzhou Institute of Zhejiang University, Huzhou 313000, China

**Keywords:** indoor localization, room impulse response, vision-assisted channel modeling, acoustic time of arrival

## Abstract

Conventional acoustic time-of-arrival (TOA) estimation in complex indoor environments is highly susceptible to multipath reflections and occlusions, resulting in unstable measurements and limited physical interpretability. This paper presents a smartphone-based indoor localization method built on vision-assisted acoustic channel modeling, and develops a fusion anchor integrating a pan–tilt–zoom (PTZ) camera and a near-ultrasonic signal transmitter to explicitly perceive indoor geometry, surface materials, and occlusion patterns. First, vision-derived priors are constructed on the anchor side based on line-of-sight reachability, orientation consistency, and directional risk, and are converted into soft anchor weights to suppress the impact of occlusion and pointing mismatch. Second, planar geometry and material cues reconstructed from camera images are used to generate probabilistic room impulse response (RIR) priors that cover the direct path and first-order reflections, where environmental uncertainty is mapped into path-dependent arrival-time variances and prior probabilities. Finally, under the RIR prior constraints, a path-wise posterior distribution is built from matched-filter outputs, and an adaptive fusion strategy is applied to switch between maximum a posteriori (MAP) and minimum mean square error (MMSE) estimators, yielding debiased TOA measurements with calibratable variances for downstream localization filters. Experiments in representative complex indoor scenarios demonstrate mean localization errors of 0.096 m and 0.115 m in static and dynamic tests, respectively, indicating improved accuracy and robustness over conventional TOA estimation.

## 1. Introduction

In applications such as personnel guidance, intelligent navigation, and emergency rescue, high-accuracy indoor positioning based on smartphones and other smart devices is of critical importance [1]. In many complex scenarios, decimeter-level or even centimeter-level positioning accuracy is required [2]. However, in indoor environments, complex architectural structures and dense infrastructure readily cause sensing signals to be blocked and reflected, making it difficult for satellite navigation systems such as global navigation satellite system (GNSS) to achieve high-precision positioning indoors [3]. At the same time, consumer-grade smart terminals exhibit large variability in sensing hardware and on-device computational capability, leading to significant fluctuations in positioning performance [4]. In addition, differences in user handset pose [5] and in the relative geometry between the terminal and sensing anchors [6] further amplify performance discrepancies. Under the constraints of not modifying smartphone hardware and maintaining low deployment costs, achieving real-time, stable, and high-accuracy positioning in complex indoor environments remains a challenging problem.

To obtain accurate user locations in complex environments, a large number of indoor positioning solutions have been proposed, including Bluetooth low energy (BLE), Wi-Fi, ultra-wideband (UWB), acoustic signals, vision, and inertial navigation [7]. In contrast, acoustic positioning does not require any additional hardware on the terminal side: the built-in microphone of a smartphone can directly receive high-precision ranging signals transmitted by anchors, offering inherent device compatibility and low deployment cost [8]. Nevertheless, indoor acoustic propagation is highly sensitive to environmental conditions. Object occlusions and multipath propagation increase the number of air-borne paths and make the direct-path component difficult to isolate [9]. Therefore, in complex environments, separating the direct acoustic path and evaluating the degree of acoustic occlusion are key steps toward improving the accuracy of high-precision acoustic positioning.

To enhance acoustic positioning accuracy, many existing studies employ multi-sensor fusion [10], high-performance hardware components [11], and data-driven correction methods [12]. These approaches alleviate, to some extent, the performance variability across terminals and improve overall system robustness. However, most acoustic correction schemes remain dominated by observational data and do not explicitly encode the physical constraints that real environments impose on acoustic propagation. Room impulse responses (RIRs) characterize how geometric structures and surface materials jointly affect the time of arrival (TOA) and energy decay of acoustic waves, thereby providing interpretable priors for direct and reflected paths [13]. By incorporating probabilistic RIRs into acoustic TOA measurements, one can impose temporal windows and energy-ordering constraints on the direct peak and propagate environmental uncertainties into ranging variance, thus improving the robustness of acoustic sensing under severe multipath and occlusion [14].

However, conventional RIR acquisition either relies on in situ measurements, which are costly and difficult to update frequently, or depends on simulation-based modeling that is sensitive to geometric and material priors and incurs high computational overhead, while deep learning approaches, although promising, remain limited in cross-scene generalization [15]. Therefore, obtaining reliable and updatable probabilistic RIR priors at low cost and effectively applying them to signal correction in acoustic sensing systems remains a key bottleneck for improving indoor acoustic localization accuracy. 

Under the constrained computational budget of smart terminals, visual priors can provide critical environmental information for analyzing acoustic propagation paths. In particular, a pan–tilt–zoom (PTZ) camera can actively capture indoor scene geometry, surface material cues, and occlusion patterns. The reconstructed environment model can then be used to generate probabilistic RIR priors that cover the direct path and first-order reflections, and to map the associated uncertainty into a calibrated variance, which serves as a physics-consistent constraint for acoustic TOA estimation [16]. However, the visual information acquired by conventional PTZ cameras is not synchronized with the propagation direction of the acoustic sensing signal. As a result, when the propagation conditions change, the corresponding RIR variations in the real environment may not be updated in time, leading to noticeable latency.

Motivated by the above considerations, this paper proposes a vision-assisted acoustic TOA optimization framework for indoor positioning. By integrating a PTZ camera and an acoustic transmitter at each anchor, the smartphone can rely solely on its built-in microphone interface to passively receive acoustic signals and anchor-side RIR information, thereby achieving signal correction and high-accuracy positioning in complex environments without modifying phone hardware. First, we design a new fusion anchor that leverages onboard images to perform semantic segmentation and geometric reconstruction of the indoor environment, extracting dominant planar structures and material attributes, and evaluating soft anchor confidence to quantify occlusion and directional risk. Second, based on the geometric and material information, we construct probabilistic RIR priors that capture direct and first-order reflections and broadcast them, together with acoustic signal parameters, from a cloud service to the smartphone. Finally, the smartphone applies matched filtering to the received acoustic signals to obtain multiple candidate TOA estimates and initial variance, and then introduces an adaptive fusion strategy (MAPSE). Under the guidance of RIR priors, MAPSE performs path posterior analysis and observation fusion, ultimately outputting TOA measurements with calibrated confidence for downstream positioning filters.

The main contributions of this paper are summarized as follows.

First, a vision–acoustic fusion anchor prototype integrating a PTZ camera and an acoustic signal transmitter is designed and implemented. A soft anchor selection method based on line-of-sight (LOS) reachability, pointing consistency, and directional risk is further proposed, providing a low-cost solution for high-accuracy indoor localization without modifying smartphone hardware.

Second, a channel modeling approach that maps visual priors to probabilistic acoustic RIR priors is developed, introducing scene geometry and material information into acoustic ranging in a physics-consistent manner and yielding calibrated priors.

Third, under the RIR prior constraint, a path-posterior analysis and adaptive fusion strategy is proposed. By switching between maximum a posteriori (MAP) and minimum mean square error (MMSE) criteria according to a posterior kurtosis threshold, robust optimization of acoustic TOA estimation in complex indoor environments is achieved, and stable high-accuracy localization under severe multipath and occlusion is validated through real-world experiments.

The remainder of this paper is organized as follows. Section 2 reviews related indoor positioning techniques and vision-assisted ranging methods. Section 3 presents the overall system design and hardware implementation. Section 4 details the generation of vision priors, probabilistic RIR modeling, and TOA posterior fusion. Section 5 describes the experimental setup and analyzes the results. Section 6 concludes the paper and outlines future research directions.

## 2. Related Work

With the widespread adoption of smartphones, indoor positioning methods targeting smart-terminal ecosystems have evolved into multiple technological routes represented by Bluetooth, Wi-Fi, UWB, acoustic signals, vision, inertial sensing, geomagnetism, and 5G, among others [17]. Table 1 compares the current mainstream indoor positioning technologies and methods for smartphone-oriented deployments. Overall, in complex indoor environments, no single-modality technique can achieve stable, high-accuracy localization for smartphones at low cost [18]. Therefore, multi-sensor fusion has become an important trend, as it leverages complementary sensing modalities to mitigate multipath interference and occlusion while maintaining practicality for consumer-grade smartphones.

In this context, acoustic localization has attracted increasing attention because it is naturally compatible with smartphone hardware, offers favorable cost–performance, and can achieve decimeter-level accuracy in typical indoor scenarios [19]. In this context, acoustic localization has attracted increasing attention because it is naturally compatible with smartphone hardware, offers favorable cost–performance, and can achieve decimeter-level accuracy in typical indoor scenarios [20]. The other direction performs peak selection and direct-path identification at the signal-processing stage to reduce false detections and missed detections [21]. Although these approaches can alleviate multipath-induced TOA errors to some extent, purely signal-domain refinements still lack explicit constraints from the physical environment.

**Table 1:** Comparison of indoor positioning methods for smartphone-oriented applications.

Positioning Method	Positioning Accuracy	Coverage Range	Deployment Cost	Maintenance Cost	Smartphone Compatibility
BLE RSSI [22]	1–3 m	10–30 m	Low	Medium	Compatible
BLE AOA [23]	0.5–1 m	1.5 × Anchor Height	Medium	Medium	Compatible
Wi-Fi RTT [24]	1–2 m	15–40 m	Medium	Low	Compatible
Wi-Fi CSI [25]	0.5–2 m	15–40 m	Medium	Low	Not Compatible
UWB TOF [26]	0.1–0.3 m	20–35 m	High	Medium	Partially Compatible
Ultrasonic TOF [27]	<0.1 m	1–5 m	High	Low	Not Compatible
Acoustic TOA [20]	0.1–0.3 m	10–40 m	Low	Low	Compatible
VIO [28]	<0.1 m	LOS	Medium	Medium	Compatible
SLAM [29]	<0.1 m	LOS	High	High	Not Compatible
IMU [30]	Accumulated	/	Low	Low	Compatible
Magnetic [31]	1–5 m	Map Coverage	Low	High	Compatible
5G NR [32]	1–3 m	Network Coverage	High	High	Partially Compatible

The RIR characterizes the full-path response observed at a receiver after an acoustic impulse emitted by a source propagates within an indoor space [33]. By analyzing RIR information, room geometry can be reconstructed to assist localization [34]. In addition, robust localization can be achieved by geometrically modeling the measured channel impulse response and combining it with machine learning to predict path parameters [35]. Overall, exploiting the environmental priors embedded in RIR is an effective way to improve localization accuracy and stability in complex indoor environments.

Conventional approaches for obtaining high-quality acoustic RIR measurements emit a known excitation signal in the target environment and record the responses using a microphone array, thereby estimating the impulse response between the device and the environment [36]. However, this typically requires relatively complex hardware setups and careful calibration. Another category synthesizes RIRs from an environment model by computing theoretical responses using priors on room geometry and surface materials, but the results are sensitive to parameter assumptions and often incur substantial computational overhead [37]. In addition, machine learning can be used to predict RIRs, where neural networks estimate reverberation characteristics, yet cross-scene generalization remains challenging [38].

To improve generality and practicality, recent studies have introduced visual sensors such as cameras to assist RIR modeling and localization. By capturing room geometry and object distributions with a camera and incorporating estimates of acoustic material properties, a predicted RIR for the environment can be constructed [39]. Visual information can also be used to identify major reflective surfaces, enabling inference of propagation distances for the direct path and mirror paths, which facilitates non-line-of-sight (NLOS) error mitigation or reflection-assisted localization [40]. However, most existing audio–visual channel modeling approaches remain limited to offline processing or simulation-based settings, and it is thus difficult for them to meet the requirements of real-time localization. In particular, it remains challenging to ensure robust generalization to unseen environments without exceeding the power and computational budgets of mobile devices.

## 3. System Architecture

### 3.1. Anchor Hardware

To improve indoor positioning accuracy under limited on-device computational resources, part of the channel-analysis workload is offloaded from the smartphone to the anchors and the cloud. To this end, a fusion anchor that integrates acoustic and visual modules is designed, enabling high-accuracy TOA estimation in typical complex indoor environments and thereby enhancing overall positioning performance.

**Figure 1:**
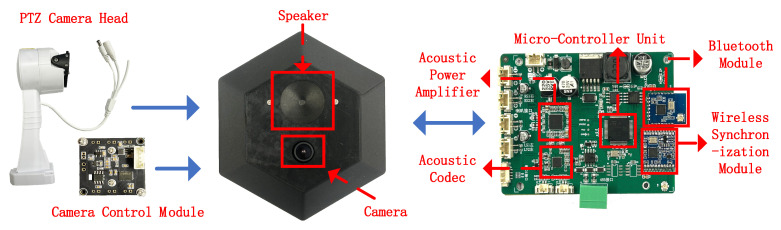
Integrated acoustic and visual module fusion anchor.

The overall structure of the anchor is shown in Figure 1 and mainly consists of the following modules: a micro-controller unit (MCU), a camera module, an acoustic codec, an acoustic power amplifier (APA), a loudspeaker, a wireless synchronization module, and a BLE interface. The MCU is responsible for task scheduling and timing management, and also performs on-board computation. The PTZ camera is used to capture environmental images, extract geometric and material priors, and carry out extrinsic calibration with respect to the acoustic transmitter. The acoustic codec and APA jointly perform digital-to-analog conversion of the swept-frequency waveform, signal shaping, and power-controlled transmission. The loudspeaker emits directional acoustic signals with known radiation patterns. The wireless synchronization module provides a common time reference for multiple anchors to achieve inter-anchor frame alignment. The BLE module is used to broadcast prior messages and receive status feedback from the smartphone.

Since the anchor must directly process captured images to perform material recognition and reflection-property estimation, the camera-side MCU in our system is equipped with convolutional inference capability at a clock frequency of 200 megahertz (MHz). This allows the anchor to run lightweight deep networks and feature compression at an update rate of 1 Hz, without significantly consuming the real-time budget reserved for acoustic TOA processing.

During each ranging cycle, the anchor uses the synchronization trigger to acquire images and compute priors, automatically controls the acoustic codec and APA to transmit the audio pulse, and then packs the prior information of candidate propagation paths—including their expected TOAs, temporal uncertainties, and path weights—into compact BLE messages that are delivered to the smartphone. These priors constrain the search space and guide the subsequent optimization of TOA estimation on the terminal side.

### 3.2. Acoustic Signal Design for Localization

Considering the bandwidth and clock-stability limitations of the audio chain on consumer smartphones, we adopt an exponential sine sweep (ESS) signal as the transmitted acoustic waveform so that both a high-quality RIR and accurate TOA estimates can be obtained within the same transmission frame. The complex baseband form of the ESS signal s(t) is given by the following:

(1) $$s(t)=exp\{j2\pi \frac{f_{0}}{\alpha }\left(e^{\alpha t}-1\right)\},\;0\leq t\leq T,$$

where $\alpha =ln(f_{1}/f_{0})/T$ is the exponential sweep rate, $f_{0}$ and $f_{1}$ denote the start and end frequencies of the sweep, respectively, and T is the sweep duration. The variable t denotes the discrete transmission-frame index, which is used to characterize the transmission, reception, and channel state associated with frame t.

The real-valued signal actually emitted into the acoustic channel is expressed as

(2) $$x\left(t\right)=w\left(t\right)\cos\left(\phi \left(t\right)\right),\;\phi (t)=(2\pi f_{0}/\alpha )\left(exp(\alpha t)-1\right)$$

where $w\left(t\right)$ is an amplitude window function and ϕ(t) is the corresponding phase.

At the smartphone side, the continuous-time received signal $r_{i,t}(u)$ for anchor i during frame t is modeled as the convolution of the transmitted signal with the linear RIR in that frame, plus additive noise:

(3) $$r_{i,t}(u)=(x_{i,t}\;*\;h_{i,t})(u)+n(u),$$

where $h_{i,t}(u)$ is the linear RIR of anchor iii in frame t, n(u) denotes additive noise, and u denotes the within-frame continuous-time independent variable, which is used to describe time-domain signals and convolution operations.

Due to possible carrier-frequency offsets and sampling-rate errors at the smartphone receiver, frequency-offset compensation and sampling correction are required. The compensated signal ${\tilde{r}}_{i,t}(u)$ is written as follows:

(4) $${\tilde{r}}_{i,t}(u)=r_{i,t}(u)exp(-j2\pi \hat{\Delta f}u),$$

where $\hat{\Delta f}$ is the online estimate of the carrier-frequency offset. This offset is obtained by linear least-squares fitting of the drift in matched-filter peak delays across adjacent frames, effectively canceling TOA drift caused by clock inaccuracy.

Guided by the prior temporal information broadcast from the anchor, the smartphone performs matched filtering and peak search within a prior time window. Specifically, it constructs a union search window:

(5) $$W_{i,t}=\underset{p}{⋃}\;\left[\tau_{i,t}^{\left(p\right)}-\kappa s_{i,t}^{\left(p\right)},\tau_{i,t}^{\left(p\right)}+\kappa s_{i,t}^{\left(p\right)}\right],$$

where $\tau_{i,t}^{\left(p\right)}$ and $s_{i,t}^{\left(p\right)}$ are the prior arrival time and standard deviation of path p, τ denotes the matched-filter delay, serving as the lag of the correlation output and the search variable for TOA candidates, respectively, and κ is a gating factor that controls the window width. When the received acoustic SNR is high and the vision-derived priors are reliable, a smaller κ is used to tighten the search window and suppress spurious peaks. Under strong occlusions or rapid motion, which reduce prior confidence, κ is moderately increased to avoid excluding the true path.

Within the union window $W_{i,t}$, the smartphone performs matched filtering between the compensated received signal and the known transmit waveform, yielding the correlation output $z_{i,t}(\tau )$:

(6) $$z_{i,t}(\tau )=\int_{W_{i,t}}\;{\tilde{r}}_{i,t}\left(u\right)s*(u-\tau )du,$$

where $s*\left(\cdot \right)$ denotes complex conjugation. The raw TOA candidate ${\hat{\tau }}_{i,t}^{raw}$ is then obtained as the delay corresponding to the maximum correlation magnitude:

(7) $${\hat{\tau }}_{i,t}^{raw}=arg\;max|z_{i,t}(\tau )|$$

By combining the anchor-side prior arrival times and temporal uncertainties, the smartphone further refines this estimate via peak interpolation and correction within the above search window. In this way, the method suppresses spurious peaks while producing time-consistent and robust TOA measurements, which in turn provide accurate inputs for subsequent positioning fusion.

### 3.3. Multiple Sensor Positioning Framework

**Figure 2:**
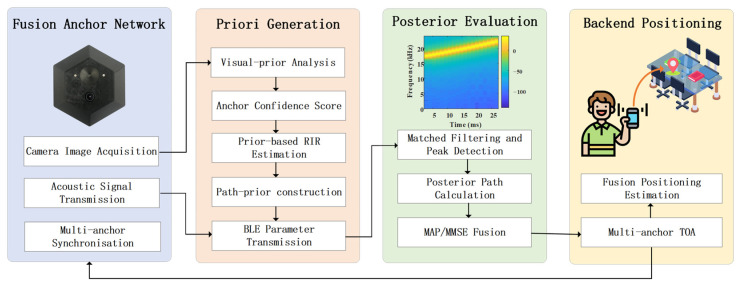
Flowchart of the proposed method.

To obtain high-accuracy TOA estimates in indoor environments with strong reverberation and severe occlusions, we construct an anchor-side fusion framework that combines vision-derived priors with acoustic ranging. As shown in Figure 2, the system generates priors at the anchor side, performs prior-constrained measurements at the smartphone side, and finally updates and feeds back the state at the back-end. The framework consists of four main modules: a fusion anchor network, a prior generation and broadcasting module, an acoustic measurement and path-posterior evaluation module, and a back-end localization module.

The fusion anchor network is composed of multiple time-synchronized fusion anchors, each integrating a PTZ camera module and an acoustic transmission module. In every ranging cycle, each anchor camera acquires environmental images and parses visible planar structures, material types, and occlusion patterns. Combined with the prior of the smartphone position from the previous cycle, the anchor derives indicators such as LOS visibility, consistency between the main lobe of the transmit beam and the anchor–phone line-of-sight direction, and direction-dependent risk induced by materials and occluders. These indicators are then aggregated into soft anchor weights. Based on this, the anchor constructs a prior set of candidate propagation paths, covering direct and first-order reflection paths, together with their temporal search windows, path-amplitude priors derived from directional and material responses, and time variances obtained by propagating geometric and extrinsic calibration uncertainties.

The prior generation module produces detailed prior information for each candidate path, including its temporal window, arrival-time variance, and weight, and encapsulates these into compact BLE messages destined for the smartphone. Each message carries a timestamp and a version number for synchronization and integrity checking. Via a low-power wireless link, these messages are delivered to the smartphone and used to constrain its correlation search and measurement formation. In multi-anchor scenarios, lightweight scheduling strategies such as time-division and frequency-division multiplexing are employed to prevent mutual interference among transmissions while preserving network-wide time synchronization.

The path-posterior evaluation module runs on the smartphone. It first compensates the received signals for frequency and sampling offsets, and then performs matched filtering, peak search, and interpolation within the prior time windows provided by the anchors, yielding raw TOA candidates and their variances. For each candidate path, the module evaluates its consistency with the observed peaks based on its temporal mean, variance, and prior weight, and thus derives a posterior probability for that path. A fusion strategy MAPASE is designed: when the candidate paths are well separable and easily distinguishable, a MAP strategy is adopted to select a single path and output its TOA and variance; in highly reverberant and occlusion-heavy conditions, where strong path competition occurs, a MMSE strategy is used instead, computing a weighted average over candidate paths with respect to their posterior probabilities. This yields smoothed and robust measurement outputs with calibrated uncertainties.

Finally, the back-end localization module takes the TOA estimates and their variances from multiple anchors as inputs to a state estimator and updates the smartphone position. Once the position estimate is obtained, the back-end feeds the smartphone position prior for the next cycle back to the anchors, enabling them to refine their vision-derived priors, temporal search windows, and path weights in a closed-loop manner.

## 4. Method

### 4.1. Vision Prior from PTZ Camera

**Figure 3:**
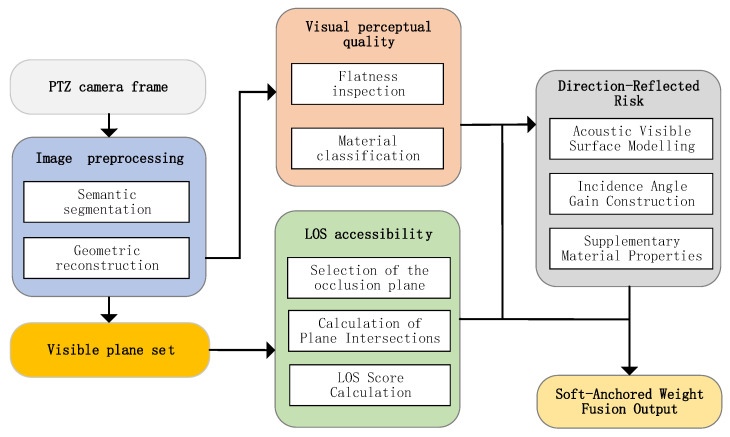
Soft anchor weight calculation.

Each anchor integrates a PTZ camera and an acoustic transmission module. On the anchor side, camera images are used to infer the geometry and planar material properties of the environment and thereby construct vision-derived priors, which in turn provide soft anchor weights for acoustic channel modeling and TOA fusion. The corresponding processing pipeline is shown in Figure 3.

Suppose that at time t, anchor $s_{i}$ acquires an image that, after semantic segmentation and geometric reconstruction, yields a set of visible planes $P_{i,t}$:

(8) $$P_{i,t}=\{(n_{j},A_{j},m_{j}){\}}_{j=1}^{K_{i}}$$

where $n_{j}$ is the unit normal vector of the j-th plane, $A_{j}$ is its effective projected area within the camera field of view, $m_{j}$ is the material label of the plane (e.g., concrete, glass, metal, wood), and $K_{i}$ is the number of planes visible to anchor i at time t.

During state estimation, the back-end fuses inertial measurements, pedestrian dead reckoning, and the previous localization result to obtain a predicted terminal position in the world coordinate frame. Based on this prediction, the unit direction vector from anchor $s_{i}$ to the terminal is defined as follows:

(9) $$d_{i,t}=\frac{{\hat{x}}_{t}-s_{i}}{∥{\hat{x}}_{t}-s_{i}{∥}_{2}}.$$

where ${\hat{x}}_{t}$ is the predicted terminal position at time t, and $∥\;{∥}_{2}$ denotes the Euclidean norm. This direction vector is used both to determine whether occluding planes exist along the line-of-sight and to quantify reflection risk in the plane–material space along that direction.

To characterize the LOS reachability between the anchor and the predicted terminal position, a subset of planes $P_{i,t}$ with occlusion semantics or with the potential to block acoustic propagation in space is selected, denoted as the occlusion-related plane set $P_{i,t}^{occ}$. This set typically includes furniture, partitions, doors, hanging structures, and similar elements. Considering the line segment $\left[s_{i},{\hat{x}}_{t}\right]$ and its intersections with planes in $P_{i,t}^{occ}$, let $C_{i,t}$ be the set of intersection points, and let $L_{c}$ be the length of the segment that traverses each occluding plane along the line direction. The LOS score for anchor i at time t is then defined as follows:

(10) $$s_{LOS,i,t}=1-\frac{\sum_{c\in C_{i,t}}\;L_{c}}{∥{\hat{x}}_{t}-s_{i}{∥}_{2}}.$$

where $s_{LOS,i,t}$ quantifies the LOS reachability between anchor iii and the predicted terminal position, and the denominator is the geometric distance between the anchor and the terminal, while the numerator is the total length of the line segment passing through occluding planes. If the line does not intersect any occluding plane, then $C_{i,t}$ is empty and $s_{LOS,i,t}=1$, indicating a completely unoccluded LOS. When the line is heavily blocked by large obstacles, the accumulated traversal length increases and $s_{LOS,i,t}$ decreases, indicating reduced LOS confidence.

**Figure 4:**
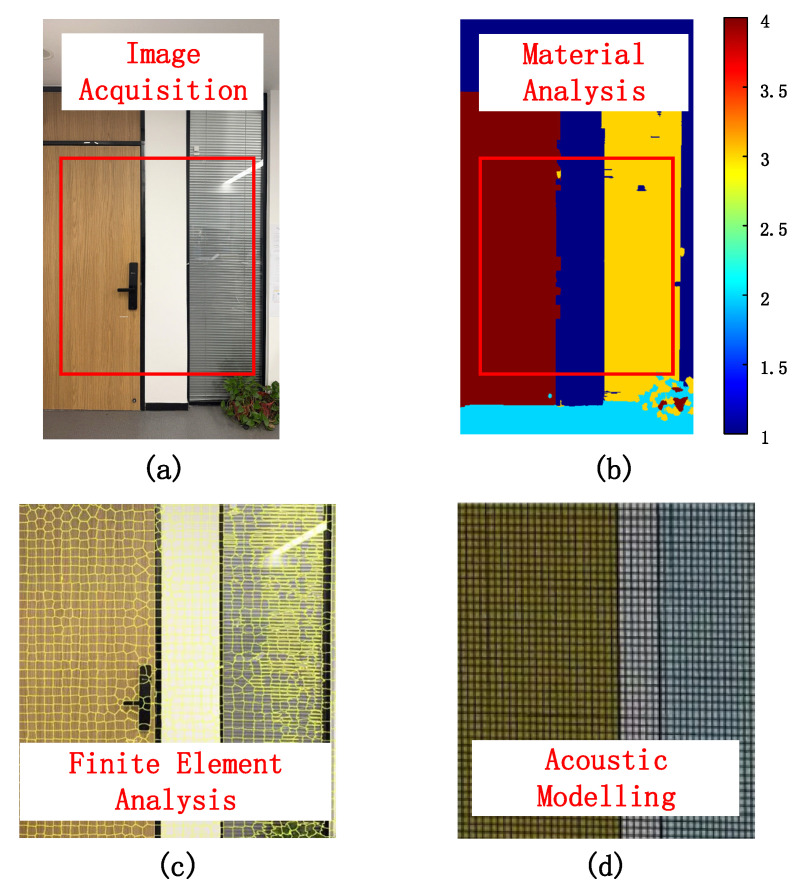
Material segmentation: (**a**) image acquisition; (**b**) material analysis; (**c**) finite element analysis; (**d**) acoustic modeling.

Figure 4 illustrates the vision-guided, material-aware acoustic channel modeling workflow. Starting from an RGB image of the indoor scene is shown in Figure 4a, an area of interest is extracted and processed by a semantic/material analysis network to obtain pixel-wise material labels such as wood, glass, and wall is shown in Figure 4b. These labeled regions are then discretized into a finite-element mesh that preserves the geometry and material boundaries of the scene is shown in Figure 4c, which serves as the basis for subsequent acoustic modeling is shown in Figure 4d, where the mesh cells are assigned material-dependent acoustic parameters and used to construct the room impulse response priors.

For reflection-risk modeling, geometric acoustics theory states that a plane can produce a significant first-order specular or quasi-specular reflection in a given direction only if its normal faces that direction. For a given propagation direction $d_{i,t}$, only consider planes whose normals form an acute angle with $d_{i,t}$ are considered, i.e., those satisfying $n_{j}^{⊤}d_{i,t}<0$. These are referred to as acoustically visible surfaces. Such surfaces contribute dominantly to reflected energy, while planes whose normals form an obtuse angle with the propagation direction lie in the “backward” half-space and have negligible contribution to the first-order reflection. Let the average absorption coefficient of material $m_{j}$ be $\alpha_{m_{j}}\in [0,1]$ and its scattering coefficient be $s_{m_{j}}\in [0,1]$. The incidence angle $\theta_{j}$ is defined via the following:

(11) $$cos\theta_{j}=-n_{j}^{⊤}d_{i,t},$$

To capture the effect of incidence angle on specular reflection strength, an incidence-angle gain function $g(\theta_{j})=|cos\;\theta_{j}|$ is introduced, which models the angular sensitivity of specular reflections: the closer the incidence direction is to the surface normal, the higher the angular gain; as the incidence angle approaches grazing, the gain tends to zero.

To incorporate material properties into directional reflection-risk modeling, an average absorption coefficient $\alpha_{m}\in [0,1]$ and a scattering coefficient $s_{m}\in [0,1]$ are preconfigured for each material type m. A larger $\alpha_{m}$ indicates stronger acoustic energy absorption, whereas a larger $s_{m}$ implies stronger scattering, which helps weaken specular reflections. By jointly considering material absorption, scattering, and incidence-angle effects, the directional reflection risk for anchor i at time t is defined as follows:

(12) $$r_{i,t}=\frac{\sum_{j:n_{j}^{⊤}d_{i,t}<0}\;A_{j}\left(1-\alpha_{m_{j}}\right)\left(1-s_{m_{j}}\right)g(\theta_{j})}{\sum_{j=1}^{K_{i}}\;A_{j}}.$$

where $r_{i,t}\in [0,1]$ is a composite reflection-risk indicator along the direction toward the terminal. The weighted sum in the numerator aggregates the potential specular reflection contributions of all acoustically visible surfaces in that direction, where the weights are jointly determined by the plane area $A_{j}$, the material’s reflection potential $\left(1-\alpha_{m_{j}}\right)$, the scattering attenuation term $\left(1-s_{m_{j}}\right)$, and the angular gain $g(\theta_{j})$. The denominator is the total area of all planes in the field of view, used for normalization. If the direction is dominated by large, smooth, low-absorption, and low-scattering hard surfaces, then $r_{i,t}$ approaches 1, indicating a high risk of strong specular multipath. Conversely, if the direction is mainly covered by highly absorptive or highly scattering materials, $r_{i,t}$ becomes small, reflecting lower reflection risk.

Since the quality of visual perception directly affects the reliability of plane–material segmentation and geometric estimation, a visual-quality score $q_{i,t}\in [0,1]$ for each anchor is further defined, obtained by normalizing and linearly combining indicators such as image sharpness, exposure adequacy, and noise level. Larger values of $q_{i,t}$ indicate more reliable visual observations for the current frame.

By combining the LOS score, directional reflection risk, and visual quality, a logistic-form soft anchor prior confidence for anchor i at time t is constructed. A linear discriminant is first defined as follows:

(13) $$z_{i,t}=\beta_{0}+\beta_{1}s_{LOS,i,t}-\beta_{2}r_{i,t}+\beta_{3}q_{i,t},$$

where $\beta_{0}$, $\beta_{1}$, $\beta_{2}$, and $\beta_{3}$ are non-negative weighting coefficients calibrated on a validation set to balance the relative importance of LOS reachability, reflection risk, and visual quality in the anchor confidence. This linear quantity is then mapped to a soft anchor weight in the interval (0,1) via the logistic function:

(14) $$w_{i,t}=\sigma (z_{i,t})=\frac{1}{1+exp(-z_{i,t})}.$$

where σ(⋅) denotes the logistic function and $w_{i,t}\in (0,1)$ is the soft prior confidence weight of anchor i at time t. A value of $w_{i,t}$ close to 1 indicates that, under the current geometric and visual conditions, the anchor is more likely to provide reliable direct-path or weak-multipath acoustic measurements and should be assigned a higher weight in subsequent RIR construction and TOA fusion. In contrast, when the LOS score is low, the reflection risk is high, and the visual quality is poor, $w_{i,t}$ automatically decreases, thereby suppressing potential NLOS and strongly reverberant anchors at the measurement level.

Through this plane–material–driven soft-anchor modeling, a quantitatively interpretable visual prior is obtained for each anchor, providing a physically meaningful and practically affordable soft-anchor confidence constraint for downstream vision-assisted acoustic channel modeling and TOA estimation.

### 4.2. RIR Construction from Vision Prior

Based on the plane–material visual priors constructed in Section 4.1, this section builds, under a geometric acoustics framework, a prior dictionary of arrival times and amplitudes for the direct path and first-order reflection paths at each anchor, and derives a probabilistic RIR model.

For anchor i and the predicted terminal position ${\hat{x}}_{t}$, the propagation time of the direct path is defined as

(15) $$\tau_{i,t}^{\left(0\right)}=\frac{∥{\hat{x}}_{t}-s_{i}{∥}_{2}}{c_{t}},$$

where $s_{i}$ denotes the spatial position of anchor i, ${\hat{x}}_{t}$ is the predicted terminal position at time t provided by the filter, and $c_{t}$ is the speed of sound under the current environmental conditions. This direct path corresponds to the line-of-sight wave between the smartphone and the anchor and serves as the reference for constructing the candidate arrival-time dictionary.

For each visible plane $\left(n_{j},A_{j},m_{j}\right)$ obtained in Section 3.1, its equation in the world coordinate system can be written as follows:

(16) $$n_{j}^{⊤}r+d_{j}=0$$

where $n_{j}$ is the unit normal vector of plane j, r is the position vector of an arbitrary point in space, and $d_{j}$ is the plane offset obtained from extrinsic calibration and geometric reconstruction, reflecting the signed displacement of the plane along its normal direction relative to the origin.

Using the image-source method, the mirror point of anchor iii with respect to plane j is defined as follows:

(17) $$s_{i}^{\left(j\right)}=s_{i}-2\left(n_{j}^{⊤}s_{i}+d_{j}\right)n_{j}.$$

Based on this mirror point, the candidate propagation time of the first-order reflection path associated with plane j is as follows:

(18) $$\tau_{i,t}^{\left(k\right)}=\frac{∥{\hat{x}}_{t}-s_{i}^{\left(j\right)}{∥}_{2}}{c_{t}}.$$

In practice, only those mirror paths whose reflection points lie within the effective region of the plane according to the geometry are retained; mirror paths that do not satisfy this condition are deemed geometrically infeasible and discarded.

By combining the direct path and all retained first-order reflection paths, the candidate TOA set for anchor i at time t is constructed as follows:

(19) $$D_{i,t}^{\tau }={\left\{\tau_{i,t}^{\left(p\right)}\right\}}_{p\in K_{i,t}},$$

where $K_{i,t}$ is the index set of retained paths, p=0 corresponds to the direct path, and p≥1 correspond to distinct first-order reflection paths. For each first-order reflection path, its geometric length can be naturally decomposed into an incident segment and a reflected segment, i.e., the distance from anchor $s_{i}$ to the reflection point plus the distance from the reflection point to the predicted terminal position ${\hat{x}}_{t}$. The sum of these two segments is uniformly denoted as $L_{i,t}^{\left(p\right)}$, representing the total propagation distance of the first-order reflection.

For amplitude modeling, the relative strength of different paths is to be distinguished without introducing excessive detailed parameters. For a path p associated with plane j, let the average absorption coefficient of material $m_{j}$ be $\alpha_{m_{j}}\in [0,1]$, the scattering coefficient be $s_{m_{j}}\in [0,1]$, and the total geometric length of the path be $L_{i,t}^{\left(p\right)}$. The amplitude factor of path p is modeled as follows:

(20) $$g_{i,t}^{\left(p\right)}=\frac{G_{0}\left(1-\alpha_{m_{j}}\right)\left(1-s_{m_{j}}\right)}{{\left(L_{i,t}^{\left(p\right)}\right)}^{\eta }},$$

where $G_{0}$ is a normalization constant that incorporates the transmit gain of the anchor and the receive gain of the smartphone, and η∈[1,2] is the distance-attenuation exponent. The direct path can be viewed as a special case in a reference medium, where $\alpha_{m_{j}}=0$ and $s_{m_{j}}=0$ are set. The amplitude of path p is denoted as $a_{i,t}^{\left(p\right)}=g_{i,t}^{\left(p\right)}$. This amplitude model differentiates the relative strength of paths through material absorption, scattering properties, and geometric propagation distance, with the goal of providing a physically reasonable strength ordering rather than reconstructing the frequency-dependent acoustic energy spectrum in detail.

The uncertainty of the arrival time is jointly determined by geometric modeling errors and the bandwidth of the transmitted signal. To keep the model simple yet physically interpretable, the TOA variance of path p is expressed as follows:

(21) $$\sigma_{\tau ,i,t}^{(p)2}=\sigma_{geom,i}^{2}+\sigma_{bw}^{2},\;\;\sigma_{bw}^{2}=\frac{C_{bw}}{B_{eff}^{2}},$$

where $\sigma_{geom,i}$ is a geometric error term for anchor iii that collectively reflects the effects of extrinsic calibration error, plane position estimation error, and state prediction error; $\sigma_{bw}^{2}$ is the lower bound on temporal resolution imposed by the effective bandwidth of the transmitted signal, $B_{eff}$ is this effective bandwidth, and $C_{bw}$ is a constant related to the signal shape and the matched-filter structure, capturing the classical inverse-square relationship between TOA estimation variance and bandwidth.

By combining the arrival time, amplitude, and temporal uncertainty of each path, the probabilistic RIR prior of anchor i at time t can be written as follows:

(22) $${\tilde{h}}_{i,t}(u)=\sum_{p\in K_{i,t}}\;a_{i,t}^{\left(p\right)}N\left(u;\tau_{i,t}^{\left(p\right)},\sigma_{\tau ,i,t}^{(p)2}\right),$$

where u is the delay variable, and $N(u;\mu ,\sigma^{2})$ denotes a one-dimensional Gaussian kernel with mean μ and variance $\sigma^{2}$. Here $\tau_{i,t}^{\left(p\right)}$, $\sigma_{\tau ,i,t}^{(p)2}$, and $a_{i,t}^{\left(p\right)}$ are the arrival time, temporal variance, and amplitude of path p, respectively. This Gaussian-mixture RIR model assigns to each physically feasible path an explicit prior on arrival time together with its uncertainty.

To inject the visual priors from Section 3.1 at the path level, the soft anchor weight $w_{i,t}$ is combined with the directional reflection-risk function to construct path prior probabilities. Let the departure direction of path p be denoted as $d_{i,t}^{\left(p\right)}$, which is the unit vector pointing from the anchor or the virtual anchor to the predicted terminal position. The corresponding directional reflection risk $r(d_{i,t}^{\left(p\right)})$ is queried from the global directional risk map using the method in Section 3.1. The unnormalized prior weight of path p is defined as follows:

(23) $${\tilde{\pi }}_{i,t}^{\left(p\right)}=w_{i,t}a_{i,t}^{\left(p\right)}exp\left(-\lambda_{r}r(d_{i,t}^{\left(p\right)})\right),$$

where $\lambda_{r}\geq 0$ is a directional-risk penalty coefficient, calibrated on an independent validation set. Specifically, a grid search is performed over $\lambda_{r}$, and the optimal value is selected by minimizing the P95 localization error. The same $\lambda_{r}$ is then fixed for all tests to ensure reproducibility.

When the propagation direction of a path has high reflection risk on the visual risk map, the term $exp\left(-\lambda_{r}r(d_{i,t}^{\left(p\right)})\right)$ significantly down-weights its prior. Normalizing over all candidate paths yields the path prior probabilities:

(24) $$\pi_{i,t}^{\left(p\right)}=\frac{{\tilde{\pi }}_{i,t}^{\left(p\right)}}{\sum_{q\in K_{i,t}}\;{\tilde{\pi }}_{i,t}^{\left(q\right)}},$$

where ${\tilde{\pi }}_{i,t}^{\left(p\right)}$ reflects the prior credibility of path p within the RIR dictionary.

Driven by the plane–material visual priors, a geometric-acoustic RIR dictionary is thus constructed for each anchor, jointly encoding path arrival times, amplitudes, uncertainties, and direction-dependent prior probabilities. This provides a physically interpretable and computationally tractable modeling foundation for the subsequent TOA fusion estimation informed by RIR priors.

### 4.3. RIR-Informed TOA Estimation

Under the constraints imposed by the above RIR priors, the smartphone constructs a Bayesian posterior over TOA candidates from the matched-filter outputs and performs bias reduction and uncertainty quantification by combining these with the path prior probabilities. An adaptive switching strategy is designed, termed MAPSE, which selects either a MAP or MMSE fusion rule based on the posterior shape and prior reliability, thereby yielding more robust TOA estimates. The corresponding procedure is summarized in Algorithm 1.

Given a local signal-to-noise ratio ${SNR}_{i,t}$ for the matched-filter output of anchor i at time t, the measurement noise variance of the raw TOA candidate can be approximated as follows:

(25) $$\sigma_{z,i,t}^{2}=\frac{1}{8\pi^{2}\beta_{2}{SNR}_{i,t}},$$

where $\beta_{2}$ is the second central-moment bandwidth of the transmit signal, which characterizes its effective spectral spread. The quantity $\sigma_{z,i,t}^{2}$ measures the temporal uncertainty of the peak location in the matched-filter output.

In the RIR priors, each physically feasible path $p\in K_{i,t}$ is characterized by an arrival-time mean $\tau_{i,t}^{\left(p\right)}$, a temporal variance $\sigma_{\tau ,i,t}^{(p)2}$, and a prior probability $\pi_{i,t}^{\left(p\right)}$. Assuming that the raw TOA candidate ${\hat{\tau }}_{i,t}^{raw}$ is triggered by one of these paths, its likelihood under path p in Gaussian noise is as follows:

(26) $$L\left({\hat{\tau }}_{i,t}^{raw}|p\right)=N\left({\hat{\tau }}_{i,t}^{raw};\tau_{i,t}^{\left(p\right)},\sigma_{\tau ,i,t}^{(p)2}+\sigma_{z,i,t}^{2}\right),$$

where $N\left(\cdot ;\mu ,\sigma^{2}\right)$ denotes the one-dimensional Gaussian density with mean μ and variance $\sigma^{2}$, and $\sigma_{\tau ,i,t}^{(p)2}+\sigma_{z,i,t}^{2}$ is the combined variance of prior timing uncertainty and measurement noise. Using the path prior probabilities $\pi_{i,t}^{\left(p\right)}$ from Section 3.2, the posterior probability of each path p given the raw TOA candidate is as follows:

(27) $$P\left(p|{\hat{\tau }}_{i,t}^{raw}\right)=\frac{N\left({\hat{\tau }}_{i,t}^{raw};\tau_{i,t}^{\left(p\right)},{\left(s_{i,t}^{\left(p\right)}\right)}^{2}+\sigma_{z,i,t}^{2}\right)\pi_{i,t}^{\left(p\right)}}{\sum_{q=0}^{K_{i}}\;N\left({\hat{\tau }}_{i,t}^{raw};\tau_{i,t}^{\left(q\right)},{\left(s_{i,t}^{\left(q\right)}\right)}^{2}+\sigma_{z,i,t}^{2}\right)\pi_{i,t}^{\left(q\right)}}.$$

This posterior $P\left(p|{\hat{\tau }}_{i,t}^{raw}\right)$ simultaneously encodes the consistency between the geometric priors of each path and the current matched-filter observation.
**Algorithm 1.** RIR-informed TOA estimation with adaptive MAP/MMSE fusion**Input: Raw TOA candidate** ${\hat{\tau }}_{i,t}^{raw}$**, Prior path parameters (**$a_{i,t}^{\left(p\right)},\;\tau_{i,t}^{\left(p\right)},\;s_{i,t}^{\left(p\right)}$**), Signal parameters** $\beta_{2}$**, Switching thresholds** $\kappa_{th},H_{th}$**, Small constant for numerical stability** ϵ**.** **Output: Fused TOA estimate** ${\hat{\tau }}_{i,t}^{fusion}$**, Associated variance** $R_{i,t}$**.** Compute the variance of the matched-filter output    **For each path** p∈{0,…,K}          Compute the total variance          Compute the likelihood of the raw TOA candidate          Compute the unnormalized posterior weight      **End For**      Normalize the posterior weights      Compute the maximum posterior probability      Sort in descending order and denote the largest and second largest as $P_{\left(i\right)}$ ($P_{\left(1\right)}$, $P_{\left(2\right)}$).      Compute the peak ratio      Compute the posterior entropy      **If** $\kappa_{i,t}\geq \kappa_{th}$ and $H_{i,t}\leq H_{th}$ % Posterior is dominated by a single peak (MAP mode)      Select the dominant path       Compute the precision terms      Fuse the raw TOA and the prior of path       Compute the associated variance       Set ${{\hat{\tau }}_{i,t}^{fusion}=\hat{\tau }}_{i,t}^{MAP}$      **Else** % Posterior is multimodal/dispersed (MMSE mode)      For each path p∈{0,…,K}      Compute precision terms      Compute the fused mean and variance for path ***p***      **End For**      Compute the MMSE estimate ${\hat{\tau }}_{i,t}^{MMSE}$ and MMSE variance $R_{i,t}^{MMSE}$      Set ${{\hat{\tau }}_{i,t}^{fusion}=\hat{\tau }}_{i,t}^{MMSE}$      **End**

In practice, the shape of the path posterior varies significantly with the scene. In LOS-dominated environments with weak reverberation, the direct path typically dominates both the priors and the observations, and the posterior is sharply unimodal. In contrast, under strong occlusion or heavy reverberation, multiple first-order reflection paths may overlap in time with the direct path, leading to a posterior with several comparable modes. To cope with this, the posterior-shape-enhanced MAPSE is proposed, which switches between MAP and MMSE estimators so as to obtain more appropriate TOA fusion results in both unimodal and multimodal regimes.

To quantify whether the posterior is dominated by a single path and to measure its dispersion, two statistics are introduced: the peak confidence and the entropy-based dispersion. The maximum posterior probability is defined as follows:

(28) $$\kappa_{i,t}=\underset{p\in K_{i,t}}{max}\;P\left(p|{\hat{\tau }}_{i,t}^{raw}\right),$$

where $\kappa_{i,t}\in [0,1]$ measures the dominance strength of the leading path in the posterior of anchor i at time t; values closer to 1 indicate a more clearly single-peaked posterior. To quantify the dispersion of the posterior over all candidate paths, the entropy is defined as follows:

(29) $$H_{i,t}=-\sum_{p=0}^{K_{i}}\;P\left(p|{\hat{\tau }}_{i,t}^{raw}\right)logP\left(p|{\hat{\tau }}_{i,t}^{raw}\right).$$

where $H_{i,t}$ measures the degree of multimodality and dispersion: larger entropy indicates that probability mass is spread more evenly over multiple paths, whereas smaller entropy implies that a few paths dominate, revealing a clearer mode structure.

Given the path posterior distribution, the MAP estimator corresponds to the optimal path decision under 0–1 loss and is suitable when the posterior is clearly unimodal and the prior on the direct path is reliable. In this case, the MAP-selected path p* is defined as follows:

(30) $$p*=arg\underset{p\in K_{i,t}}{max}\;P\left(p|{\hat{\tau }}_{i,t}^{raw}\right),$$

When the MAP fusion strategy is activated, the raw TOA candidate and the prior of the dominant path p* are fused as two Gaussian sources, yielding the MAP-refined TOA estimate with associated confidence variance:

(31) $${\hat{\tau }}_{i,t}^{MAP}=\frac{{\hat{\tau }}_{i,t}^{raw}\sigma_{z,i,t}^{-2}+\tau_{i,t}^{\left(p*\right)}{\left(s_{i,t}^{\left(p*\right)}\right)}^{-2}}{\sigma_{z,i,t}^{-2}+{\left(s_{i,t}^{\left(p*\right)}\right)}^{-2}},.$$

This variance $R_{i,t}^{MAP}={\left(\sigma_{z,i,t}^{-2}+{\left(s_{i,t}^{\left(p*\right)}\right)}^{-2}\right)}^{-1}$ represents the reliability of the TOA measurement under the single-dominant-path assumption; smaller values indicate more reliable measurements.

When the posterior is clearly multimodal and dispersed, forcing a pure MAP decision on a single path tends to underestimate the true uncertainty. In such cases, an MMSE fusion strategy is adopted that marginalizes over all paths with probability weighting, thereby yielding a more reasonable TOA estimate in the mean-square-error sense. Specifically, for each path p, the raw candidate and its prior are first fused as two Gaussians, obtaining a path-wise posterior mean $\mu_{i,t}^{\left(p\right)}$ and variance $\Sigma_{i,t}^{\left(p\right)}$:

(32) $$\mu_{i,t}^{\left(p\right)}=\frac{{\hat{\tau }}_{i,t}^{raw}\sigma_{z,i,t}^{-2}+\tau_{i,t}^{\left(p\right)}{\left(s_{i,t}^{\left(p\right)}\right)}^{-2}}{\sigma_{z,i,t}^{-2}+{\left(s_{i,t}^{\left(p\right)}\right)}^{-2}},$$

(33) $$\Sigma_{i,t}^{\left(p\right)}={\left(\sigma_{z,i,t}^{-2}+{\left(s_{i,t}^{\left(p\right)}\right)}^{-2}\right)}^{-1},$$

The overall MMSE TOA estimate is computed by averaging over all paths with posterior probabilities as weights:

(34) $${\hat{\tau }}_{i,t}^{MMSE}=\sum_{p=0}^{K}\;P\left(p|{\hat{\tau }}_{i,t}^{raw}\right)\mu_{i,t}^{\left(p\right)}$$

The corresponding MMSE confidence variance is as follows:

(35) $$R_{i,t}^{MMSE}=\sum_{p=0}^{K}\;P\left(p|{\hat{\tau }}_{i,t}^{raw}\right)\left(\Sigma_{i,t}^{\left(p\right)}+{\left(\mu_{i,t}^{\left(p\right)}-{\hat{\tau }}_{i,t}^{MMSE}\right)}^{2}\right)$$

Based on the above statistics, the MAP–MMSE adaptive switching rule in MAPSE is defined as

(36) $${\hat{\tau }}_{i,t}^{fusion}=\left\{\begin{array}{cc} {\hat{\tau }}_{i,t}^{MAP}, & if\;\kappa_{i,t}\geq \kappa_{th}\;and\;H_{i,t}\leq H_{th},\\ {\hat{\tau }}_{i,t}^{MMSE}, & otherwise. \end{array}\right.$$

where $\kappa_{th}$ and $H_{th}$ are thresholds for the maximum posterior probability and the posterior entropy, respectively, obtained from offline calibration data. When the posterior is clearly dominated by a single path and the entropy is low, MAPSE selects the MAP fusion rule to achieve stronger outlier suppression and smaller variance. When the posterior is more dispersed and evidently multimodal, MAPSE automatically switches to MMSE fusion to avoid overconfident estimates and reduce the overall mean-square error.

The TOA measurement model used in the back-end localization filter is thus

(37) $$y_{i,t}={\hat{\tau }}_{i,t}^{fusion}=\frac{∥x_{t}-s_{i}{∥}_{2}}{c_{t}}+b_{i}+\varepsilon_{i,t}.$$

where $x_{t}$ is the terminal position to be estimated, $b_{i}$ is the clock-offset-induced bias of anchor i, and $\varepsilon_{i,t}∼N(0,R_{i,t})$ is zero-mean Gaussian noise with variance $R_{i,t}$ (chosen as $R_{i,t}^{MAP}$ or $R_{i,t}^{MMSE}$ according to the active mode). By performing MAPSE adaptive fusion between the matched-filter peak and the vision-informed priors, the proposed method strongly suppresses NLOS outliers when the direct path is dominant, while preserving realistic uncertainty in multipath-rich conditions, thereby significantly improving both the accuracy and robustness of the overall positioning system. 

## 5. Experiment

### 5.1. Experimental Setup

To validate the positioning performance of the proposed audio–visual fusion system, experiments were conducted in two representative indoor environments: a typical office and a long corridor.

**Figure 5:**
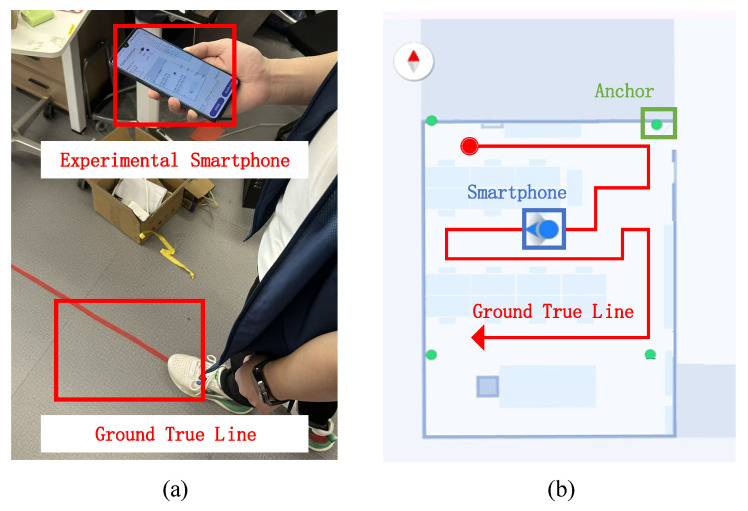
Walking along a predetermined ground true line while holding a mobile phone: (**a**) ground true line and experimental smartphone; (**b**) phone display.

In Scenario 1 (office), a typical office environment measuring approximately 10.33 m in length and 7.87 m in width was selected. Using an 01dB sound level meter, the reverberation time RT60 of this scene was measured to be 0.5 s, which corresponds to a medium-reverberation localization area. A Xiaomi 11 smartphone, as shown in Figure 5a, was used to evaluate the proposed method. The device runs Android 14 and is equipped with a Qualcomm Snapdragon 888 processor and 8 GB of random-access memory (RAM), representing a mid-range commercial off-the-shelf (COTS) terminal. Prior to the experiment, red adhesive tape was placed on the floor to mark the ground-truth trajectory. A test subject walked along this predefined route while running the developed smartphone application for real-time positioning, as shown in Figure 5b.

To ensure real-time execution on smartphones, the operating system should support audio acquisition and BLE communication, the mobile CPU should be capable of running the required computations in real time, and at least 4 GB of RAM is needed to buffer audio frames and intermediate variables so that real-time TOA estimation and localization outputs can be produced on-device. Since environmental perception does not rely on the smartphone camera, no strict requirement is imposed on camera imaging quality.

**Figure 6:**
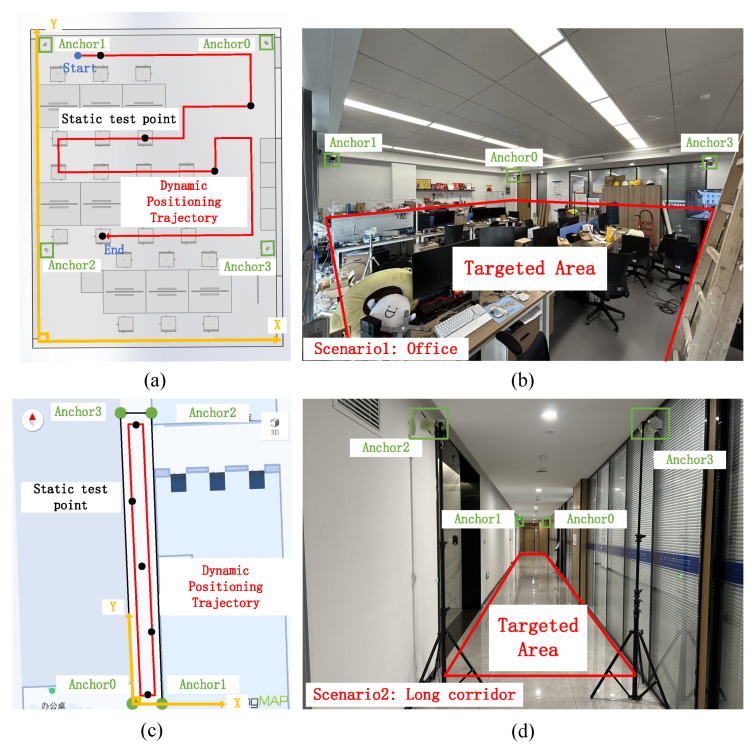
Experimental setup: (**a**) schematic of office deployment; (**b**) actual office scene; (**c**) schematic of long corridor deployment; (**d**) actual long corridor scene.

In the office experiment of Scenario 1, four fusion anchors (indexed 0–3) were deployed, as shown in Figure 6a, where each anchor is highlighted by a green rectangle. Black solid dots indicate the points used for static positioning experiments. The feasible walking area and predefined ground-truth trajectory are shown in Figure 6b, where the red polyline represents the reference path from the starting blue solid dot to the static test points. A laser rangefinder and measuring tape were used to precisely survey the anchor locations. Taking the lower-left corner of the room as the origin, with the x-axis pointing to the right and the y-axis pointing upward, a local coordinate system was established. In this system, the coordinates of anchors 0, 1, 2, and 3 are (7.088, 9.895, 2.531), (0.303, 9.895, 2.351), (0.303, 2.519, 2.513), and (7.088, 2.519, 2.513), respectively.

To further assess the robustness of the proposed system in strongly multipath-rich environments, an additional experiment was conducted in Scenario 2 (long corridor). The corridor is approximately 20.00 m in length and 1.68 m in width. Using the same 01dB sound level meter, the reverberation time RT60 was measured as 0.8 s, corresponding to a highly reverberant localization area. The smartphone model and application used in Scenario 2 were identical to those in the office experiment. As shown in Figure 6c, four fusion anchors (0–3) were deployed in the corridor and are indicated by green solid circles; black solid dots again represent static test points. The feasible walking area is overlaid with a red line indicating the predefined ground-truth trajectory as shown in Figure 6c. In this scenario, anchor 0 was chosen as the origin of the coordinate system, with the x-axis pointing to the right and the y-axis pointing upward. In this coordinate frame, the coordinates of anchors 0, 1, 2, and 3 are (0, 0, 2.52), (0.303, 9.895, 2.52), (0.303, 2.519, 2.52), and (7.088, 2.519, 2.52), respectively.

**Figure 7:**
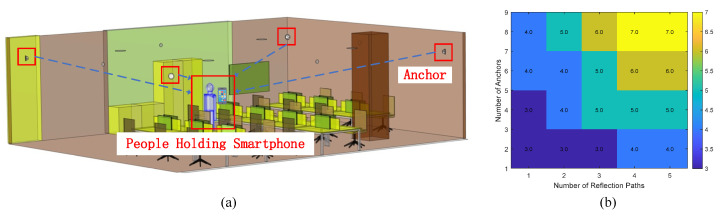
Real-time analysis: (**a**) environmental diagram; (**b**) detailed timing breakdown.

To evaluate the practicality of the proposed system under realistic deployment conditions, as shown in Figure 7a, the end-to-end computational cost was further analyzed as a function of the number of anchors and the number of effective reflection paths per anchor. Specifically, the number of anchors and the maximum number of effective reflection paths were varied, and the total runtime of four key modules was measured: ESS matched filtering, RIR prior construction, posterior computation and MAPSE switching, and positioning fusion. As shown in Figure 7b, the results show that, for configurations with 2–8 anchors and 1–5 effective paths per anchor, a complete environment-prior reconstruction cycle—including image acquisition, material analysis, RIR structure update, and prior broadcasting—requires approximately 3–7 s, and increases smoothly with system scale. This observation is consistent with the expected slightly super-linear growth in computational complexity with respect to the number of anchors and reflection paths.

It is important to emphasize that this time cost corresponds to the low-frequency prior-update process at the anchor side, rather than the per-frame computation in the smartphone localization loop. During online localization, the smartphone simply reads the cached probabilistic RIR priors and performs millisecond-level TOA estimation and fusion updates; the per-frame latency remains well below 20 ms. Consequently, even under the most complex configuration with 8 anchors and 5 reflection paths per anchor, prior reconstruction in the cloud can be scheduled at low frequency in the background with caching, without violating the real-time constraints of the smartphone-side processing. These results indicate that, at typical deployment scales, the proposed audio–visual fusion framework can simultaneously satisfy both accuracy and real-time requirements.

### 5.2. Ablation Performance Analysis

To systematically evaluate the practical contributions of the proposed vision-assisted probabilistic RIR priors and the MAPSE switching strategy, a set of ablation studies is conducted by constructing multiple TOA estimation and localization configurations for comparative analysis. In total, five methods are considered:

- M0 (Acoustic-only): a baseline that relies solely on acoustic measurements;
- M1 (Vision-gating): M0 augmented with coarse time-window constraints derived from visual geometry;
- M2 (RIR+MAP): method using probabilistic RIR priors with a fixed MAP fusion strategy (no adaptive switching);
- M3 (RIR+MMSE): method using probabilistic RIR priors with a fixed MMSE fusion strategy;
- M4 (Proposed): the full method combining vision-assisted probabilistic RIR priors and the MAPSE adaptive switching mechanism.

Both static and dynamic experiments are performed to quantify the marginal contributions of each module to positioning accuracy and robustness.

The data for M0 were collected under non-occluded conditions to characterize the ideal upper-bound performance of the acoustic TOA baseline. In contrast, the data for M1 to M4 were collected with dynamic occlusions introduced, while keeping the same trajectory and parameter settings, in order to quantify the gains of visual priors and probabilistic RIR constraints on TOA stability and localization robustness under occlusion disturbances. Furthermore, although furniture movements and variations in pedestrian flow were present, their impact on the dominant propagation paths and channel modeling results was relatively limited. Therefore, this experiment mainly evaluates the system response speed and localization stability during environmental changes.

In the static experiment, five reference points are selected in the office scenario to cover diverse geometric and occlusion conditions, including LOS points near anchors, points partially occluded by desks or chairs, and strongly reflective points near glass or walls. For each reference point, collect approximately 60 frames of localization results are continuously collected under stable system operation. At each frame, all five methods M0–M4 are evaluated in parallel, yielding a total of 300 static samples. The aggregated results are summarized in Table 2.

To comprehensively characterize localization reliability under severe multipath and occlusion, the mean error is reported together with the cumulative distribution function (CDF) of localization errors. The CDF represents the cumulative proportion of samples whose errors do not exceed a given threshold. A higher curve at the same error threshold indicates that a larger fraction of localization results falls within that threshold, and thus the CDF provides an intuitive measure of localization stability and reliability in complex indoor environments.

**Figure 8:**
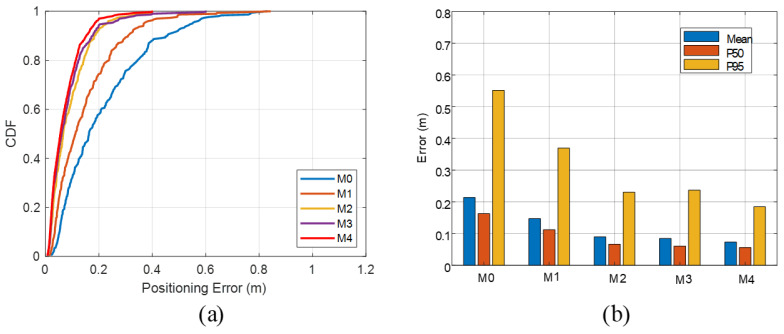
The static localization ablation results: (**a**) the CDFs of positioning error; (**b**) the RMSE comparison across methods.

As shown in Figure 8a, The pure acoustic baseline M0 achieves an average positioning error of 0.214 m, a 50th percentile (P50) error of 0.163 m, a 95th percentile (P95) error of 0.551 m, a maximum error of 0.831 m, and an RMSE of 0.270 m, exhibiting a clearly heavy-tailed error distribution. After introducing coarse time-window constraints from visual geometry, M1 reduces the static mean error to 0.147 m, while P50 and P95 drop to 0.112 m and 0.370 m, respectively, and RMSE decreases to 0.193 m. Compared with M0, this corresponds to roughly 30% reductions in both mean error and RMSE, and about a 33% reduction in P95, indicating that even coarse visual gating of TOA can suppress a substantial portion of severe outliers. However, the maximum error for M1 remains around 0.84 m, on the same order as M0, implying that time-window constraints alone cannot completely prevent the selection of extreme erroneous peaks.

Building on M1, the introduction of vision-assisted probabilistic RIR priors in M2 and M3 yields further consistent improvements in the static scenario. For M2, the mean error is 0.090 m, P50 is 0.066 m, P95 is 0.230 m, and RMSE is 0.116 m. Relative to M1, the mean error and RMSE are further reduced by about 39% and 40%, respectively, and P95 decreases by around 38%, while the maximum error shrinks to 0.402 m. The fixed-MMSE variant M3 achieves slightly better mean and P50 errors than M2, with mean and P50 reduced to 0.085 m and 0.061 m, respectively. However, its P95 and RMSE are broadly comparable to, or slightly worse than, those of M2, and the maximum error increases to 0.603 m. This suggests that, under multimodal posterior distributions, MMSE tends to yield smaller overall bias and smoother estimates, while MAP retains an advantage in suppressing the most extreme error peaks.

The full configuration M4 achieves the best overall static performance. Its mean error is reduced to 0.073 m, the P50 error to 0.056 m, P95 to 0.185 m, and RMSE to 0.096 m, with the maximum error kept at 0.402 m, identical to that of M2. Compared with the acoustic-only baseline M0, M4 reduces the mean, P95, and RMSE by approximately 66%, 66%, and 64%, respectively. Relative to the RIR-only methods M2/M3, M4 further tightens the mean, P50, P95, and RMSE across the board, the mean error is reduced by about 19% compared with M2 and by roughly 14% compared with M3, without sacrificing the maximum error. These results indicate that the posterior-shape-driven MAPSE adaptive switching in MAPSE can automatically select more suitable estimators between LOS-dominated unimodal cases and strongly multipath multimodal cases, thereby preserving the long-tail suppression capability provided by the RIR priors while further improving overall accuracy and the stability of the error distribution.

**Figure 9:**
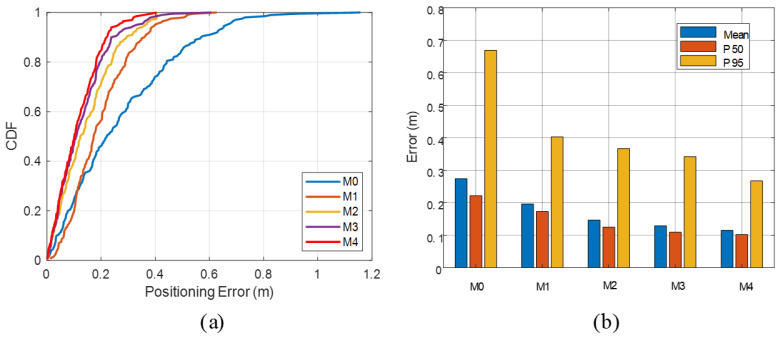
The dynamic localization ablation results: (**a**) the CDFs of positioning error; (**b**) the RMSE comparison across methods.

In the dynamic trajectory experiment, 200 localization frames are collected along a predefined walking path. As shown in Figure 9, the acoustic-only baseline M0 yields a dynamic mean error of 0.274 m, P50 0.222 m, P95 0.669 m, maximum error 1.157 m, and RMSE 0.346 m. In complex multipath environments, this pure acoustic TOA solution exhibits poor trajectory continuity and frequent error spikes. Introducing visual time-window constraints in M1 substantially compresses the dynamic mean error to 0.196 m, reduces P95 to 0.403 m, and lowers RMSE to 0.228 m. Compared with M0, this corresponds to reductions of about 28%, 40%, and 34% in the mean, P95, and RMSE, respectively, while the maximum error drops to 0.627 m, demonstrating that visual geometric filtering of outliers remains effective in dynamic scenarios.

Leveraging the vision-assisted RIR priors, M2 and M3 further improve the dynamic performance. For M2, the mean error, P50, P95, and RMSE are 0.147 m, 0.125 m, 0.367 m, and 0.180 m, respectively. Relative to M1, this represents additional reductions of about 25% in mean error and 21% in RMSE, with a further ≈9% improvement in P95, and the maximum error is compressed to 0.405 m. The MMSE-based variant M3 (RIR+MMSE) attains a mean error, P50, P95, and RMSE of 0.129 m, 0.109 m, 0.342 m, and 0.164 m, respectively, outperforming M2 on mean-type statistics and P95, but at the cost of a larger maximum error of 0.608 m. This again confirms that MMSE offers better average convergence under short-term multipath-induced drifts along the trajectory, whereas MAP remains slightly superior in suppressing occasional extreme deviations.

The method M4 also delivers the best overall performance in the dynamic scenario. The experimental results show that M4 achieves a mean error of 0.115 m, P50 0.102 m, P95 0.267 m, and RMSE 0.142 m, while keeping the maximum error within 0.405 m, consistent with M2. Compared with the baseline M0, M4 reduces the mean, P95, and RMSE by about 58%, 60%, and 59%, respectively. Relative to the vision-gated method M1, the mean error and RMSE are further lowered by approximately 41% and 38%. Compared with the RIR-only configurations M2 and M3, M4 still achieves 10–27% improvements on mean-type metrics and P95. The statistics for the entire trajectory are summarized in Table 3.

Taken together, the static and dynamic experiments demonstrate that the vision-assisted probabilistic RIR priors substantially enhance the reliability of acoustic TOA estimation in complex multipath environments, while the posterior-shape–based MAPSE adaptive switching enables fine-grained selection between MAP and MMSE strategies. As a result, the proposed localization framework consistently outperforms all baselines in terms of both accuracy and robustness.

### 5.3. Static Ranging Performance

**Figure 10:**
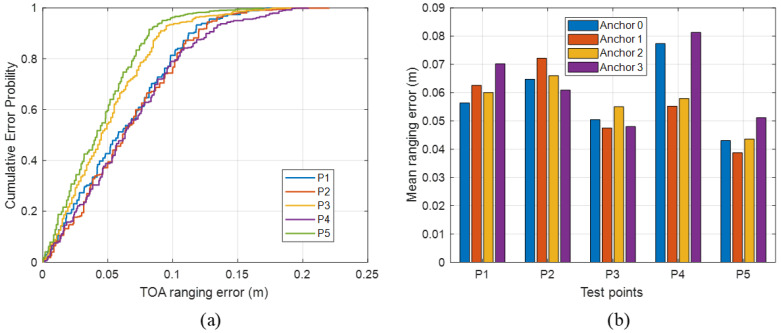
Ranging performance in the office scenario: (**a**) CDF of ranging error; (**b**) RMSE of TOA ranging error.

In the office scenario, TOA range measurements between the smartphone and the four anchors were collected at five static test locations, and the corresponding ranging errors were statistically analyzed. The CDF curves of the ranging error are shown in Figure 10a. Across all anchors, the mean ranging errors fall within 0.044–0.068 m, P50 errors are within 0.041–0.065 m, and P95 errors lie in the range of 0.091–0.149 m. Figure 10b plots the RMSE of the TOA ranging errors between the smartphone and the four anchors at the five static test points. The overall RMSE of the proposed system is approximately 0.049 m, demonstrating that the system achieves consistently high-accuracy ranging performance in a complex office environment and maintains robustness in a realistic indoor setting with moderate reverberation and occlusions. Detailed data on distance measurement errors are shown in Table 4.

### 5.4. Static Positioning Performance

**Figure 11:**
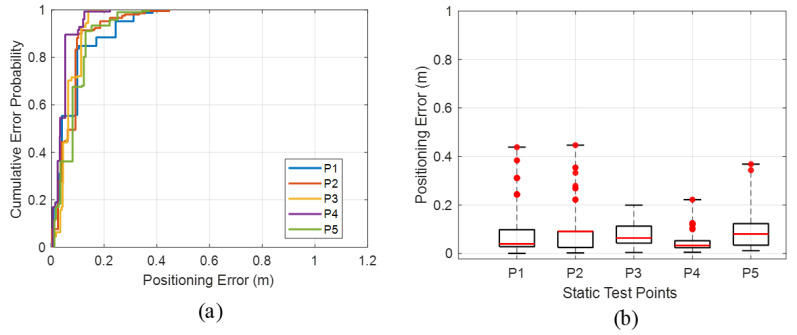
Static positioning performance in the office scenario: (**a**) CDF of static positioning error; (**b**) comparison of static positioning errors.

In the office scenario, the cumulative distribution of static positioning error is shown in Figure 11a. For the proposed system, the average 50th-percentile, 95th-percentile, and maximum positioning errors across all static test points are 0.061 m, 0.182 m, and 0.335 m, respectively, and the overall mean positioning error is 0.096 m. These results clearly demonstrate that the system provides high-accuracy localization performance in the office environment.

Figure 11b reports the statistical distribution of static positioning errors in the office scenario, including the maximum and minimum values, lower quartile (Q1), upper quartile (Q3), P50 (red line), and outliers (red dots). In Scenario 1, the smartphone positioning error ranges from 0.003 m to 0.447 m, and P50 error is 0.052 m, indicating that the system can deliver highly accurate localization for the majority of test points in this scene. A detailed comparison of static positioning statistics in the office scenario is summarized in Table 5.

**Figure 12:**
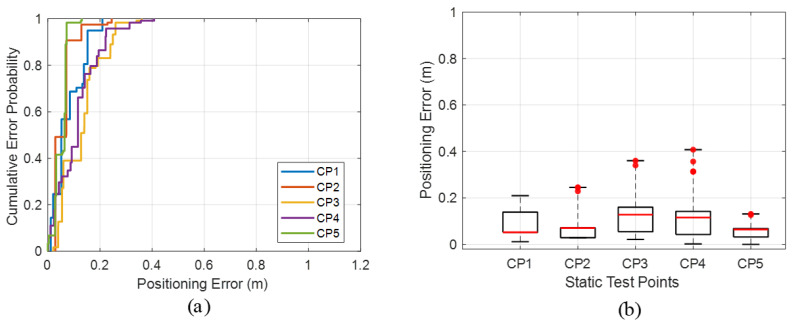
Experimental results in the corridor scenario: (**a**) CDF of static positioning error; (**b**) comparison of static positioning errors.

In the long-corridor scenario, the cumulative distribution of static positioning error is shown in Figure 12a. The proposed system achieves average P50, P95, and maximum positioning errors of 0.086 m, 0.174 m, and 0.271 m, respectively, with an overall mean positioning error of 0.109 m. These results confirm that the system also maintains strong localization performance in the corridor environment.

Figure 12b shows the statistical results of the localization experiment in the long-corridor scenario. In Scenario 2, the smartphone positioning error ranges from 0.001 m to 0.408 m, and the P50 error is 0.068 m, indicating that the system can provide highly accurate localization for the majority of test points in this environment. Detailed numerical results for this test are summarized and comprehensively compared in Table 6.

### 5.5. Dynamic Positioning Performance

**Figure 13:**
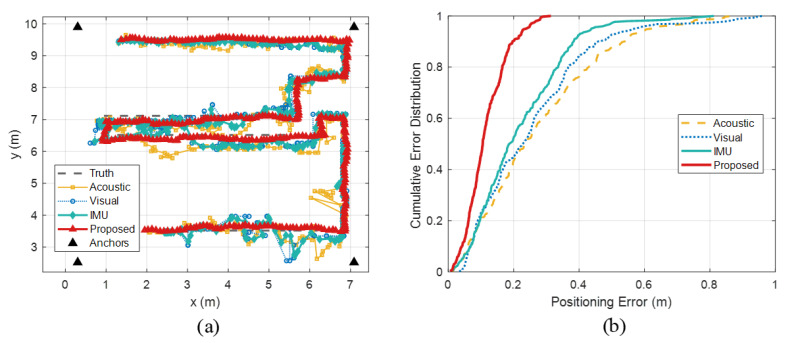
Experimental results in the corridor scenario: (**a**) CDF of static positioning error; (**b**) comparison of static positioning errors.

In the office scenario, a dynamic localization test was conducted using a smartphone, as shown in Figure 13a. In the figure, the black filled triangles (Anchors) denote the localization anchors, and the black dashed line (Truth) indicates the ground-truth trajectory. The yellow curve (Acoustic) shows the acoustic TOA trajectory, where delay estimation and localization are performed using GCC-PHAT [41]. The blue curve (Visual) corresponds to the vision-based trajectory, where CHENG [42] is adopted with fixed camera anchors for localization. The green curve (IMU) represents the acoustic TOA trajectory fused with IMU information, where WANG [43] fuses acoustic measurements with pedestrian dead reckoning to obtain the localization result. The red solid curve (Proposed) shows the trajectory produced by the proposed method, and the same notation is used hereafter. It can be observed that the proposed method maintains a continuous and smooth trajectory in regions where single-modality methods are strongly affected by multipath interference, and closely follows the ground-truth path, particularly when the user’s moving direction is nearly parallel to the anchor–user line-of-sight.

Figure 13b further quantifies performance by comparing the cumulative distribution of positioning errors for different methods along the trajectory. The proposed method achieves P50 and P95 positioning errors of 0.105 m and 0.240 m, respectively. In contrast, the acoustic TOA method yields P50 and P95 errors of 0.235 m and 0.625 m, the vision-only method achieves 0.229 m and 0.574 m, and the acoustic-TOA+IMU fusion method attains 0.188 m and 0.440 m for the P50 and P95. The advantage of the proposed approach is more pronounced in regions with strong multipath and environmental changes. Traditional fusion schemes tend to exhibit larger errors in dynamic scenarios, whereas the proposed method exploits prior RIR information to suppress multipath, effectively reducing measurement bias and significantly improving long-term tracking stability. Detailed numerical results for this test are summarized and compared in Table 7.

**Figure 14:**
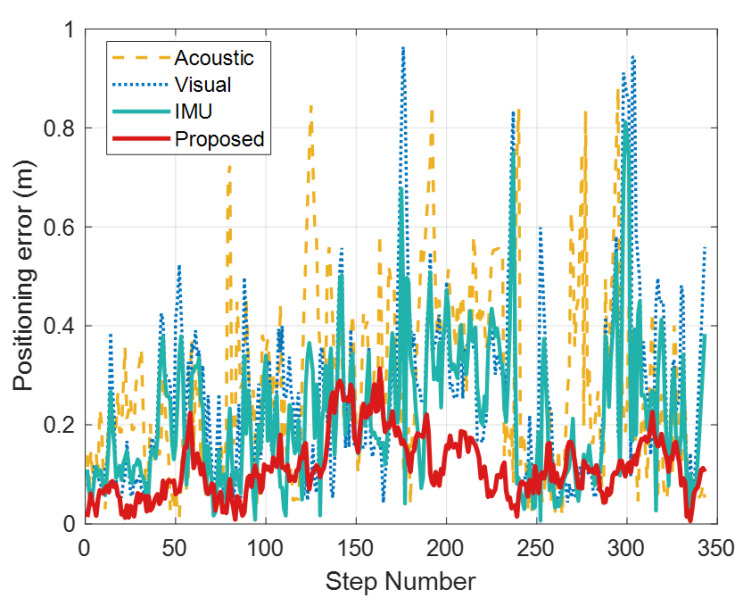
Comparison of positioning errors under different positioning methods.

Figure 14 shows the error variations in the proposed method and the baseline methods along the dynamic localization trajectory. The time evolution of the localization error provides an indicator of stability and robustness for dynamic tracking. The error of the proposed algorithm ranges from 0.006 m to 0.314 m, whereas the acoustic TOA, vision-only, and acoustic-TOA+IMU methods exhibit much larger fluctuations, with error ranges of 0.033–0.964 m, 0.013–0.881 m, and 0.007–0.812 m, respectively. These results indicate that the proposed method not only achieves higher accuracy than conventional fusion approaches in large-scale dynamic indoor environments, but also constrains the fluctuation of positioning error within approximately 0.308 m, representing improvements of about 67%, 65%, and 62% over the acoustic TOA, vision-only, and acoustic-TOA+IMU methods, respectively. This demonstrates the clear advantage of the proposed framework in achieving high-precision and robust localization under complex indoor conditions.

**Figure 15:**
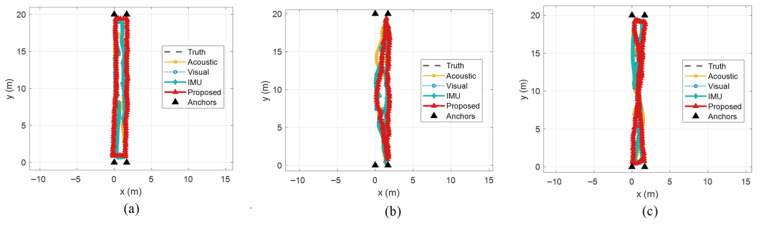
The dynamic positioning trajectory in long corridor (**a**) rectangular; (**b**) triangular; (**c**) hourglass-shaped.

In the office scenario, the feasible area for localization is relatively fixed. To further verify the robustness of the proposed fusion framework under different deployment conditions, the dynamic positioning performance was additionally evaluated in a long-corridor scenario. A test subject walked along three predefined trajectories while holding the smartphone, namely a rectangular trajectory, a triangular trajectory, and an inverted-triangle trajectory, as shown in Figure 15a–c, respectively. As before, black solid triangles denote the anchors, the black dashed lines represent the ground-truth trajectories, the yellow curves correspond to the acoustic TOA-only trajectories, the blue curves to the vision-based trajectories, the green curves to the acoustic-TOA+IMU fusion trajectories, and the red solid curves to the trajectories estimated by the proposed method. It can be seen that, even in the corridor with pronounced multipath, the proposed method maintains continuous and smooth trajectories that closely adhere to the ground truth, particularly when the user’s motion direction is nearly parallel to the anchor–terminal baselines.

**Figure 16:**
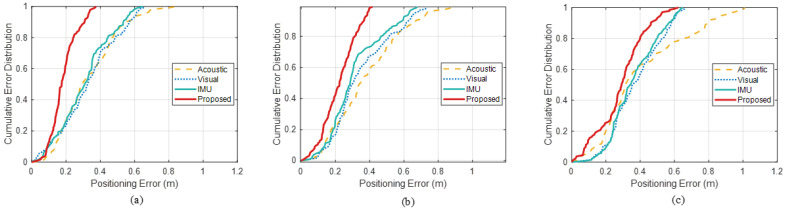
The dynamic positioning trajectory in long corridor (**a**) rectangular; (**b**) triangular; (**c**) hourglass-shaped.

To further quantify performance, Figure 16 reports the cumulative error distributions for different methods over the three corridor trajectories. Averaged over the three trajectories, the proposed method achieves P50 and P95 positioning errors of 0.230 m and 0.414 m, respectively. In comparison, the acoustic TOA method attains 0.321 m and 0.764 m, the vision-only method 0.331 m and 0.630 m, and the acoustic-TOA+IMU fusion method 0.273 m and 0.602 m at P50 and P95. In this highly multipath corridor environment, all three baselines exhibit pronounced error fluctuations, whereas the proposed method, by leveraging prior RIR information to suppress multipath, produces significantly smaller variations in measurement error and maintains robust localization performance.

Across the three paths, the proposed algorithm achieves an average positioning error of 0.232 m, while the acoustic TOA, vision-only, and acoustic-TOA+IMU methods attain average errors of 0.375 m, 0.353 m, and 0.331 m, respectively. This corresponds to improvements of approximately 38%, 34%, and 30% over the three baselines. These results show that, compared with traditional fusion schemes, the proposed method delivers more accurate and more robust localization in narrow, strongly multipath indoor environments such as long corridors. Detailed statistics for this test are also summarized and compared in Table 8.

### 5.6. Efficiency Performance

Table 9 compares the proposed cloud-assisted, vision-guided RIR feedback scheme with several representative enhancement strategies in terms of typical indoor accuracy, on-device computation, and energy cost. Among these, the LFM+GCC method was employed as the reference for comparison. The conventional LFM+GCC baseline provides a very light-weight implementation but suffers from 0.5 to 1.0 m P50 and pronounced long-tail errors under strong multipath. In contrast, the proposed method improves P50 to about 0.3–0.6 m and markedly reduces P95 and outage probability, while keeping the computation and energy on the smartphone within approximately 1–1.2 of the baseline, since most of the heavy RIR modeling is offloaded to the cloud. Other approaches such as FRFT-based super-resolution, multi-sensor fusion, and on-device deep learning can achieve comparable or slightly better accuracy, but typically require 3–10 higher computation and 2–8 higher energy consumption. This comparison highlights that the proposed scheme achieves a substantially better accuracy–complexity trade-off and is therefore more suitable for large-scale deployment on passenger smartphones.

## 6. Conclusions

A smartphone-based indoor positioning method is presented based on vision-assisted acoustic channel modeling for high-accuracy TOA estimation under severe multipath and occlusion. A fusion anchor integrating a PTZ camera and a near-ultrasonic signal transmitter is developed, together with a vision-driven probabilistic RIR modeling scheme and an RIR-informed adaptive TOA fusion framework. Scene geometry, surface materials, and occlusion patterns are leveraged to construct RIR priors that cover the direct path and first-order reflection paths. MAPSE is then applied to adaptively switch between MAP and MMSE estimators, producing debiased TOA measurements with calibratable variances. Experiments conducted with a commercial smartphone demonstrate mean positioning errors of 0.096 m in static tests and 0.115 m in dynamic tests, consistently outperforming the acoustic baseline, the visual baseline, and the acoustic TOA fused with an IMU baseline in both accuracy and robustness. These results indicate strong practicality and promising scalability for high-precision indoor positioning in smartphone ecosystems. It should be noted that the framework remains dependent on the visibility and calibration quality of anchor-side visual priors, and prior updates may degrade under severe occlusion or rapid scene changes. Future work will investigate fast online updating and view scheduling with multi-anchor collaboration to further improve stability and deployment scalability in complex dynamic environments.

## Figures and Tables

**Table 2:** The static localization ablation results.

Method	Mean (m)	Max (m)	RMSE (m)	P50 (m)	P95 (m)
M0	0.214	0.831	0.270	0.163	0.551
M1	0.147	0.843	0.193	0.112	0.370
M2	0.090	0.402	0.116	0.066	0.230
M3	0.085	0.603	0.119	0.061	0.237
M4	0.073	0.402	0.096	0.056	0.185

**Table 3:** The dynamic localization ablation results.

Method	Mean (m)	Max (m)	RMSE (m)	P50 (m)	P95 (m)
M0	0.214	0.831	0.270	0.163	0.551
M1	0.147	0.843	0.193	0.112	0.370
M2	0.090	0.402	0.116	0.066	0.230
M3	0.085	0.603	0.119	0.061	0.237
M4	0.073	0.402	0.096	0.056	0.185

**Table 4:** The comparison of ranging measurement error.

Static Points	P1	P2	P3	P4	P5
Mean (m)	0.062	0.066	0.050	0.068	0.044
P50 (m)	0.060	0.061	0.048	0.065	0.041
P95 (m)	0.125	0.130	0.108	0.149	0.091

**Table 5:** Comparison of static positioning errors in the office scenarios.

Static Points	P1	P2	P3	P4	P5
Mean (m)	0.082	0.075	0.068	0.044	0.094
Max (m)	0.439	0.447	0.199	0.222	0.368
P50 (m)	0.040	0.091	0.063	0.033	0.080
P95 (m)	0.244	0.185	0.140	0.120	0.221

**Table 6:** Comparison of static positioning errors in the corridor scenarios.

Static Points	CP1	CP2	CP3	CP4	CP5
Mean (m)	0.081	0.058	0.130	0.116	0.051
Max (m)	0.209	0.245	0.360	0.408	0.131
P50 (m)	0.052	0.071	0.128	0.116	0.064
P95 (m)	0.187	0.129	0.260	0.224	0.072

**Table 7:** Comparison of dynamic positioning error in the office scenario.

Method	Acoustic	Visual	IMU	Proposed
Mean (m)	0.274	0.255	0.218	0.115
P50 (m)	0.235	0.229	0.188	0.105
P95 (m)	0.625	0.574	0.440	0.240

**Table 8:** Comparison of dynamic positioning error in the corridor scenario.

Trajectory	Error	Acoustic	Visual	IMU	Proposed
Rectangular	Mean (m)	0.334	0.330	0.310	0.186
	P50 (m)	0.298	0.328	0.188	0.175
	P95 (m)	0.657	0.611	0.572	0.332
Triangle	Mean (m)	0.385	0.353	0.318	0.223
	P50 (m)	0.348	0.305	0.286	0.224
	P95 (m)	0.736	0.670	0.631	0.388
Hourglass-shaped	Mean (m)	0.407	0.377	0.364	0.288
	P50 (m)	0.317	0.359	0.345	0.291
	P95 (m)	0.898	0.609	0.604	0.521

**Table 9:** Comparison of on-device cost and accuracy between the proposed method and representative enhancement schemes.

Method Class	Typical Indoor Accuracy (P50)	On-Device Computation	Extra Energy on Smartphone
GCC-PHAT	0.5–1.0 m	1× (reference)	1× (reference)
FRFT [44]	0.2–0.5 m	3–5× (reference)	2–4× (reference)
WANG	0.4–1.0 m	2–4× (reference)	2–3× (reference)
Deep learning [45]	0.2–0.7 m	5–10× (reference)	3–8× (reference)
Proposed	0.3–0.6 m	1–1.2× (reference)	≈1× (reference)

## Data Availability

The original contributions presented in the study are included in the article; further inquiries can be directed to the corresponding author.

## Abbreviations and Nomenclature

**Abbreviations**		
APA	Acoustic Power Amplifier	
BLE	Bluetooth Low Energy	
CDF	Cumulative Distribution Function	
COTS	Commercial Off-The-Shelf	
CPU	Central Processing Unit	
ESS	Exponential Sine Sweep	
FRFT	Fractional Fourier Transform	
GCC	Generalized Cross-Correlation	
GCC-PHAT	Generalized Cross-Correlation with Phase Transform	
GNSS	Global Navigation Satellite System	
IMU	Inertial Measurement Unit	
LFM	Linear Frequency Modulation	
LOS	Line-of-Sight	
MAP	Maximum a Posteriori	
MAPSE	MAP–MMSE Adaptive Switching Estimator	
MCU	Microcontroller Unit	
MMSE	Minimum Mean Square Error	
NLOS	Non-Line of Sight	
P50	50th Percentile	
P95	95th Percentile	
PTZ	Pan–Tilt–Zoom	
RAM	Random Access Memory	
RGB	Red–Green–Blue	
RIR	Room Impulse Response	
RMSE	Root Mean Square Error	
RT60	Reverberation Time (RT60)	
SNR	Signal-to-Noise Ratio	
TOA	Time of Arrival	
UWB	Ultra-Wideband	
Wi-Fi	Wireless Fidelity	
**Nomenclature**		
**Symbol**		**D** **efinition**
i		Anchor index.
t		Discrete transmission frame index.
u		Continuous time variable inside a frame.
τ		Delay variable in matched filtering.
p		Index of a propagation path.
s(t)		Complex baseband form of the ESS.
x(t)		Real acoustic waveform transmitted into the channel.
w(t)		Amplitude window applied to the transmitted waveform.
ϕ(t)		Instantaneous phase of the transmitted waveform.
$f_{0}$		Start frequency of the ESS.
$f_{1}$		End frequency of the ESS.
T		Duration of one sweep.
α		Exponential sweep rate parameter of ESS.
$r_{i,t}(u)$		Received signal associated with anchor *i* in frame *t*
$h_{i,t}(u)$		RIR between anchor *i* and the smartphone in frame *t*.
n(u)		Additive noise in the received signal.
$\hat{\Delta f}$		Estimated carrier frequency offset used for compensation.
${\tilde{r}}_{i,t}(u)$		Compensated received signal.
$W_{i,t}$		Union search window.
$\tau_{i,t}^{\left(p\right)}$		Prior arrival time of path *p* for anchor *i* in frame *t*.
$s_{i,t}^{\left(p\right)}$		Prior timing uncertainty of path p.
κ		Gating coefficient.
$z_{i,t}(\tau )$		Matched-filter output as a function of the delay variable.
${\hat{\tau }}_{i,t}^{raw}$		Raw TOA candidate selected from the matched-filter peak.
$s_{LOS,i,t}$		Line of Sight reachability score for anchor *i* in frame *t*.
$q_{i,t}$		Visual quality score.
r(d)		Directional reflection risk value.
$w_{i,t}$		Soft anchor weight
$d_{i,t}^{\left(p\right)}$		Unit direction vector of path p.
$a_{i,t}^{\left(p\right)}$		Path amplitude used in the probabilistic RIR prior.
${\tilde{\pi }}_{i,t}^{\left(p\right)}$		Unnormalized prior weight of path p.
$\pi_{i,t}^{\left(p\right)}$		Normalized prior probability of path p.
$\lambda_{r}$		Directional risk penalty coefficient.
$\rho_{i,t}^{\left(p\right)}$		Posterior probability of path p.
$\kappa_{i,t}$		Peak confidence score.
$H_{i,t}$		Entropy of the posterior distribution describing dispersion.
$\kappa_{th}$		Threshold for peak confidence in the switching rule.
$H_{th}$		Threshold for posterior entropy in the switching rule.
${\hat{\tau }}_{i,t}$		Final fused TOA estimate.
$R_{i,t}$		Variance associated with the fused TOA estimate.
$s_{i}$		Anchor position in the global coordinate system.
$x_{t}$		Smartphone position to be estimated in frame t.
$c_{t}$		Speed of sound
$b_{i}$		Anchor-dependent timing bias term in the TOA model.
$\varepsilon_{i,t}$		Random measurement noise term in the TOA model.
